# Dysregulation of sphingolipid metabolism contributes to the pathogenesis of chronic myeloid leukemia

**DOI:** 10.1038/s41419-025-07594-0

**Published:** 2025-04-13

**Authors:** Yinyin Xie, Qinghua Zeng, Zhiwei Chen, Jiachun Song, Fuhui Wang, Dan Liu, Xiaojian Sun, Yuanliang Zhang, Qiuhua Huang

**Affiliations:** 1 Shanghai Institute of Hematology, State Key Laboratory of Medical Genomics, National Research Center for Translational Medicine at Shanghai, Ruijin Hospital Affiliated to Shanghai Jiao Tong University School of Medicine, Shanghai, China; 2 Jiangxi Provincial Key Laboratory of Hematological Diseases, Department of Hematology, The First Affiliated Hospital, Jiangxi Medical College, Nanchang University, Nanchang, Jiangxi China; 3 Department of Pathophysiology, Key Laboratory of Cell Differentiation and Apoptosis, Chinese Ministry of Education, Shanghai Jiao Tong University School of Medicine, Shanghai, China

**Keywords:** Experimental models of disease, Chronic myeloid leukaemia

## Abstract

Chronic myeloid leukemia (CML) is primarily driven by the BCR::ABL1 oncoprotein, which has potent tyrosine kinase activity. BCR::ABL1 has been shown to facilitate several metabolic processes, including glycolysis, lipid synthesis, and protein synthesis in vitro. However, the altered metabolic profile in vivo remains poorly understood. Using Scl/tTA-*BCR::ABL1* mice as a model, we conducted an analysis of plasma metabolites at different stages following *BCR::ABL1* induction. Metabolites involved in sphingolipid and thiamine metabolism were significantly altered at the early stage of CML, while the tricarboxylic acid (TCA) cycle metabolites were altered during disease progression. Among these metabolic changes, sphingolipid metabolism is of particular significance. Inhibition of sphingolipid metabolism had a more pronounced effect on the growth and survival fate of K562 cells compared to thiamine metabolism inhibition. Furthermore, knockdown of sphingosine kinase 1 (SPHK1) resulted in extensive metabolic remodeling, affecting lipid, energy, and heme metabolism. Pharmacological targeting of sphingolipid metabolism appeared to attenuate the development of CML. Our study also demonstrated that BCR::ABL1 triggers ERK-dependent phosphorylation of SphK1, leading to aberrant activation of sphingolipid metabolism, which in turn has a positive feedback effect on BCR/ABL expression. These findings highlight the dominant role of sphingolipid metabolism in BCR::ABL1-induced metabolic reprogramming in CML.

## Introduction

Cells have the ability to adapt their metabolic pathways to maintain cellular homeostasis and meet the varying demands of growth in the context of environmental stress. This phenomenon is known as “metabolic reprogramming” [1]. A number of oncogene-related signaling pathways, including AMPK, PI3K-AKT-mTOR, and RAS-RAF-MEK-ERK, have been shown to play a role in initiating of metabolic reprogramming [2]. The Warburg effect, in which tumor cells rely more on aerobic glycolysis than on oxidative phosphorylation like normal cells, has been widely recognized as a major hallmark of tumor cell metabolic reprogramming for over a century [3]. However, the development of metabolism-based anti-cancer drugs has been limited in the last decade, and further elucidation of the underlying metabolic mechanisms of tumor cells is required [4].

Chronic myeloid leukemia (CML) is a myeloproliferative neoplasm that arises from hematopoietic stem cells. It is characterized by a reciprocal translocation between chromosomes 9 and 22, also known as the Philadelphia chromosome, resulting in the production of the BCR::ABL1 oncoprotein with potent tyrosine kinase activity [5, 6]. Previous research has shown that *BCR::ABL1* induces concomitant biological processes through the activation of multiple signaling pathways, including but not limited to the PI3K-AKT-mTOR and RAS-RAF-MEK-ERK pathways [7]. These pathways display a high degree of overlap with metabolic signaling pathways. The presence of *BCR::ABL1* results in abnormalities in cellular glucose metabolism and transport processes, and can lead to the production of increased levels of reactive oxygen species via the PI3K/mTOR pathway [8–10]. The activity of sphingomyelin synthase 1 in lipid metabolism has been shown to be affected by *BCR::ABL1* [11]. Branched-chain amino acid (BCAA) metabolism is also reprogrammed by *BCR::ABL1* [12]. In addition, tyrosine kinase inhibitors (TKIs), represented by imatinib (STI571), are effective drugs for the treatment of CML [13]. Their efficacy and resistance mechanisms have been shown to be closely linked to alterations in metabolic properties [14, 15].

Sphingolipids are bioactive molecules that play a pivotal role in regulating cancer cell signaling, thereby controlling tumor suppression and survival. The metabolic network of sphingolipids provides regulatory nodes that control cancer growth and/or proliferation in response to cellular stress. This includes the activation of enzymes that generate the tumor suppressor ceramide and/or inhibit the conversion of ceramide to sphingosine 1 phosphate (S1P) or other complex sphingolipids [16, 17]. S1P biosynthesis is mediated by sphingosine kinases 1 and 2 (SPHK1 & SPHK2), of which SPHK1 is cytoplasmic and SPHK2 is nuclear. Both have shown potential for use in tumor therapy in the context of hematological malignancies [18, 19]. BCR::ABL1 has been shown to upregulate *SPHK1* expression through a number of signaling pathways, including the MAPK, PI3K, and JAK2 pathways [20]. Overexpression of *SPHK1* is regulated by signaling through PI3K, AKT2, and mTOR in imatinib-resistant CML cells [21]. In addition, SPHK1 functions as a downstream regulator of imatinib-induced apoptosis and plays a key role in the TKI resistance mechanism in CML cells [22, 23].

Current studies on the metabolic characteristics of CML cells have mainly been performed in vitro, and the altered metabolic profile in vivo remains poorly understood. In this study, using the widely accepted Tet-off-induced transgenic CML mice (Scl/tTA-*BCR::ABL1*) as a model [24], we analyzed significant changes in the metabolic profile at differeny stages following *BCR::ABL1* induction and demonstrated that sphingolipid metabolism plays a particularly critical role in the pathogenesis of CML.

## Materials and methods

### Animal models

Scl/tTA-*BCR::ABL1* double transgenic mice were routinely maintained with 200 μg/mL doxycycline (Dox) (Clontech, USA) in drinking water. Dox was withdrawn from 4 week–old mice to induce *BCR::ABL1* expression.

### Plasma collection and metabolomics analysis

Plasma samples were collected at different time points following *BCR::ABL1* induction (0, 1, and 3 weeks). Each 100 μL plama sample was mixed with 400 μL methanol containing 0.02 mg/mL internal standard in a centrifuge tube for extraction. The supernatant was used for the following analysis. Metabolite profiling was conducted using LC-MS/MS. A pooled quality control sample was prepared by mixing an equal volumes of each plasma sample. Data were processed and analyzed using Progenesis QI software, and metabolites were identified using the HMDB and Metlin databases. PCA and OPLS-DA analyses was performed using the R package “ropls”. Significant metabolites were identified based on VIP values and *p*-values. Metabolites with VIP > 1 and *p* < 0.05 were considered significantly different based on OPLS-DA and *t*-test. Differential metabolites were analyzed using the KEGG database and the Python package “scipy.stats” for pathway mapping.

### Cell culture

32D cells were cultured in RPMI 1640 medium (Gibco, USA) supplemented with 10% fetal bovine serum and 10% IL-3 WEHI-3B conditioned medium. K562 cells were cultured in RPMI 1640 with 10% fetal bovine serum (Gibco, USA). Cells were maintained at 37 °C with 5% CO2 tested for mycoplasma contamination.

### Cell line construction

32Dcl3 (32D) cells were infected with retroviral supernatants of MigR1-*BCR::ABL1*-IRES-mCherry or Migr1-IRES-mCherry, respectively, in the presence of polybrene (Sigma-Aldrich, USA). mCherry^+^ cells were isolated by flow cytometry after 48 h and re-sorted after one week to generate stable cell lines expressing *BCR::ABL1*-IRES-mCherry (32D-BA) or mCherry alone (32D-MigR1).

### shRNA knockdown

K562 cells were infected by pLKO-sh-SPHK1 virus supernatants. After 48 h, 1 mg/ml puromycin (Beyotime, China) was added in culture medium for 3 days to select the stable cell line. Knockdown efficiency was assessed by qRT-PCR.Target and scramble sequences shRNA are as follows: *SPHK1*-shRNA1:GCAGCTTCCTTGAACCATTAT,*SPHK1*-shRNA1-scramble:ATCGTGGACTCTGACAGATCCG;*SPHK1*-shRNA2:ACCTAGAGAGTGAGAAGTATC,*SPHK1*-shRNA2-scramble:GACGAGTTCGAGTCCATGATTG;*TPK1*-shRNA1:GGATGTGAGCTCATTTCAACT,*TPK1*-shRNA1-scramble:GAACTTTTGGAGCATCATTG;*TPK1-*shRNA2:CATTGGTCAGTACTTCCAATA,*TPK1*-shRNA2-scramble:TTACTTGGACATCAATTAGTC.

### Drug Treatment

K562 cells with SPHK1 or TPK1 knockdown were seeded in cell culture dishes at a density of 2 × 10^6^ cells/ml and then treated with different drugs for 48 h (Z-VAD-FMK, 10 μM, HY-16658B, MCE; Necrostatin-1, 1 μM, HY-15760, MCE). 32D-BA cells were seeded in cell culture dishes at a density of 3×10^6^ cells/ml and then treated with different drugs for 48 h (Imatinib, 10 μM, HY-15463, MCE; Dastinib, 1 μM, HY-10181, MCE; ASN007, 1Μm, HY-136579, MCE; ERK inhibitor 7, 1 μM, HY-142433, MCE; FTY720, 10 nM, HY-12005, MCE; PF543, 1 μM, HY-15425, MCE). K562 cells were seeded in cell culture dishes at a density of 2 × 10^6^ cells/ml and then treated with different S1PRs inhibitor for 48 h (S1PR1 inhibitor W146 TFA, 10 μM, HY-101395A, MCE; S1PR2 inhibitor JTE013, 1 μM, S7182,Selleck; S1PR3 inhibitor CAY10444, 5 μM, E1158, Selleck; S1PR4 inhibitor CAY50358), 0.1 uM, HY-136462, MCE; S1PR1/5 inhibitor Siponimod, 0.1 uM, S7179, Selleck)

### Western blotting

An aliquot of 1 × 10 ^ 7 cells was lysed with 1 ml of 1 × SDS loading buffer (P0015, Beyotime) and boiled at 100°C for 10 min. The resulting samples were centrifuged at 12, 000 rpm for 10 min at 4 °C, and the supernatant was used for Western blotting. 15 ul of the supernatant was separated by SDS-polyacrylamide gel electrophoresis and electrotransferred to polyvinylidene difluoride (PVDF) membranes (ISEQ00010, Sigma, Darmstadt, Germany), followed by overnight incubation at 4°C with primary antibodies(SPHK1,ab302714,Abcam;p-SPHK1,AF8318,Affinity;SPHK2,ab320741,Abcam;

p-SPHK2,AF3532,Affinity; ERK, ab184699, Abcam; p-ERK, ab201015, Abcam; mTOR, ab134903; Abcam; p-mTOR, ab109268, Abcam; BCR::ABL1, ab187831, Abcam; AKT, ab8805, Abcam; p-AKT, ab38449; Abcam; SGPL1, DF13867, Affinity; SGPP1, DF13902, Affinity; SGPP2, PA5-42767, Thermofisher; β-ACTIN, AF7018, Affinity). Active bands were detected using horseradish peroxidase-conjugated secondary antibodies (401353, 401253, Millipore, Burlington, Massachusetts, USA) and Immobilon Western Chemiluminescent HRP Substrate (WBKLS, Sigma) on the ImageQuantLAS4000 system (General Electric Company).

### Bulk RNA-seq analysis

Total RNA was extracted from the cell line using TRIzol® reagent according to the manufacturer’s instructions. RNA quality was assayed using the 5300 Bioanalyser (Agilent, USA) and quantified using the ND-2000 (NanoDrop Tech, USA). The RNA-seq library was prepared with Illumina® Stranded mRNA Prep, Ligation from Illumina (San Diego, CA), using 1ug of total RNA. RNA-seq data were analyzed by first checking the quality of raw reads using FastQC and then mapping clean reads to the mouse reference genome using Hisat2. Transcript expression levels were calculated using TPM, and gene abundance was quantified using RSEM. Differential expression analysis was performed using DESeq2, identifying significantly differentially expressed genes with |log2FC | ≧ 1 and FDR < 0.05. Functional-enrichment analysis was conducted to identify significantly enriched DEGs in GO terms and metabolic pathways with a Bonferroni-corrected *P*-value < 0.05 compared to the whole transcriptome background, using Goatools for GO analysis and Python scipy for KEGG analysis.

### Flow cytometric analysis and cell sorting

Bone marrow cells were collected from mice with chronic myeloid leukemia (CML) and then stained in phosphate-buffered saline (PBS) at 4 °C for 30 min with specific antibodies to isolate cells of different lineages, including erythroid progenitor cells (CD45-, Ter119 + , CD71 + ), myeloid cells (CD45 + , GR1 + , MAC1 + ), and lymphoid cells (CD45 + , B220 + , MAC1-). Antibodies used were obtained from Biolegend. Flow cytometric sorting experiments were performed using a BD FACSAriaTM instrument from BD Biosciences.

### Quantitative Real-Time Polymerase Chain Reaction (qRT-PCR)

Total RNA was extracted using TRIzol reagent (Invitrogen, Carlsbad, CA) according to the manufacturer’s instructions. First-strand cDNA synthesis was performed using PrimerScript RT Master Mix (RR036A, Takara Bio, Shiga, Japan), and quantitative real-time polymerase chain reaction (qRT-PCR) was performed using TB Green Premix Ex Taq (RR420A, Takara Bio) on the ViiA 7 Real-Time PCR platform (Life Technologies). GAPDH was used as an internal control, and each sample was analyzed in triplicate. Melting curve analysis validated the specificity of the amplification. Relative mRNA expression levels compared to GAPDH were determined using the 2^-ΔΔCt^ method.

### Erythroid colony-forming unit assay

Clonogenic erythroid progenitor cells were quantified using a methylcellulose colony assay medium with or without erythropoietin (EPO) (MethoCult™ SF M3434, Stem Cell Technologies). Specifically, 1 × 10^4^ total bone marrow cells from mice with chronic myeloid leukemia (CML) and mice with reduced sphingosine kinase 1 (SK1-KD) were plated in 35-mm tissue culture dishes containing the colony assay medium. After incubation for 7 days at 37 °C in 5% CO2, erythroid CFU (E) colonies were enumerated under an inverted microscope.

### Viral preparation and bone marrow transplantation

293 T cells were transfected with the pMSCV-BA-IRES-mCherry plasmid accompanied by EcoPack using Lipofectamine 2000 (Invitrogen, USA) as per the manufacturer’s protocol. Viral supernatant was collected 48 h after transfection. Donor mice (Balb/c) were treated with 5-fluorouracil 5 days before harvest of BM from tibias and femurs. BM cells were infected by the pMSCV-BA-IRES mCherry virus twice. 1 × 10^6^ total infected cells were resuspended in 200 μL PBS and injected into the tail vein of each lethally irradiated recipient (female Balb/c). For survival analysis, recipient mice were treated with different drugs on day 12 post-transplantation. Briefly, recipient mice were randomly assigned to four groups (*n* = 11 per group), and each group received daily oral gavage as follows: PBS solution (control group), imatinib solution (100 mg/kg/day; i.g.), PF543 solution (10 mg/kg/day; i.p.) or FTY720 solution (10 mg/kg/day; i.p.), each at a volume of 100 µL per mouse daily. Drug administration was continued until the PBS-treated mice died. The date on which each mouse died was recorded for survival analysis.

### Statistical analysis

The Student’s *t*-test and Pearson correlation coefficient were used to analyze the differences between groups using GraphPad Prism for Windows version 9.5.1 (GraphPad Software). A *p*-value (*p*) <0.05, was considered statistically significant.

## Results

### Altered metabolic profile in CML mice

**Fig. 1:**
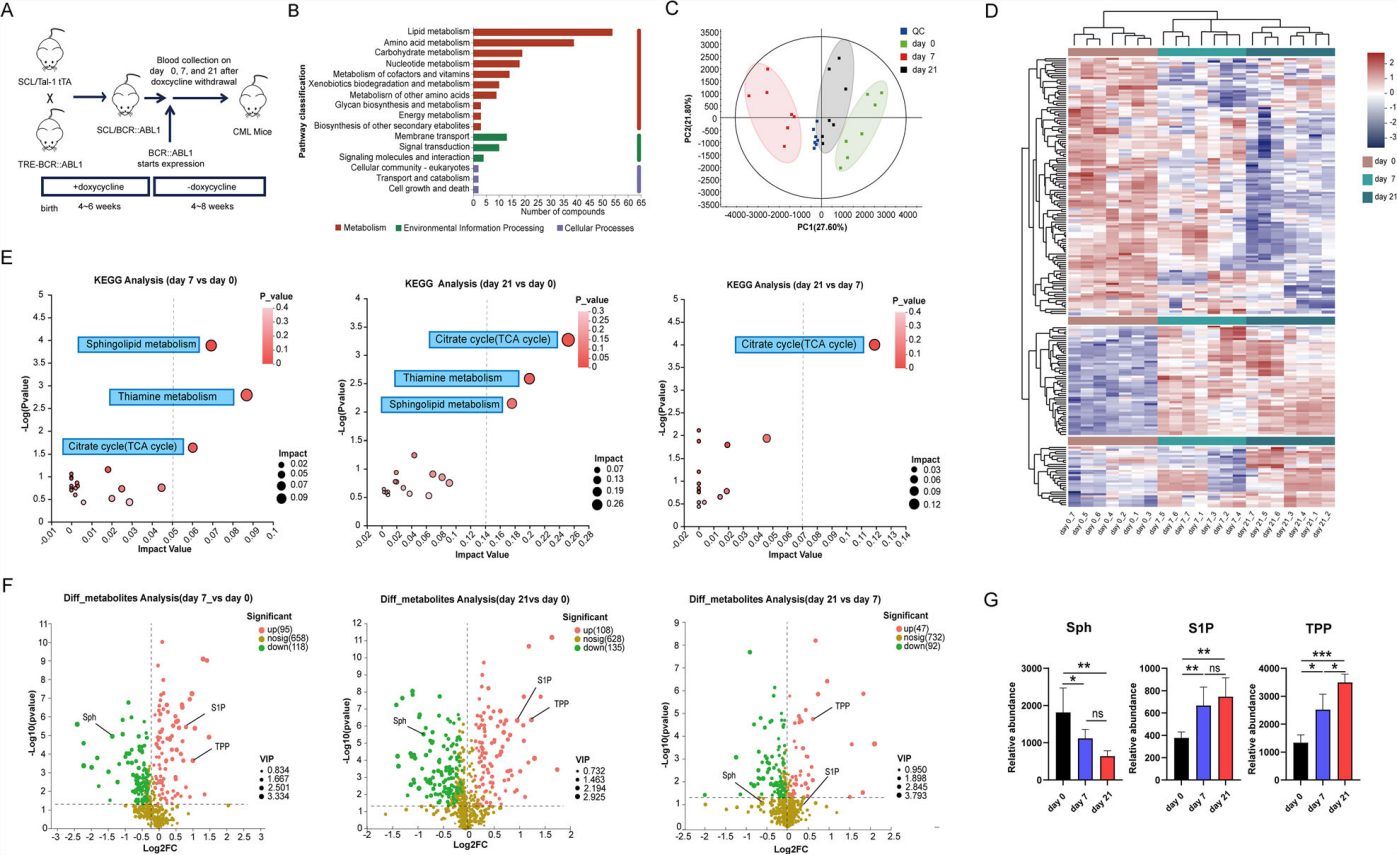
Untargeted metabolomic analysis of the plasma from Scl/tTA-*BCR::ABL1* mice. **A** Schematic diagram illustrating the sample collection for untargeted metabolomics (*n* = 7). **B** KEGG pathway classification of metabolites identified by untargeted metabolomics. **C** Principal component analysis of different groups subjected to metabolomics. **D** Heatmap analysis depicting the top 200 metabolites identified by untargeted metabolomics. **E** Topology plot of KEGG pathway analysis with differential metabolites between different groups. **F** Volcano plot illustrating differential metabolites between different groups (VIP ≥ 1 and *p*-value ≤ 0.05). **G** Quantitative mass spectrometry validation of differential metabolites (*n* = 5). The bar graph data in Fig. 1 are presented as mean ± S.E.M.

Previous studies have shown that Scl/tTA-*BCR::ABL1* mice exhibit clear CML characteristics within 4–8 weeks following doxycycline withdrawal, with a median survival rate of approximately 56 days [25]. To investigate the alterations in the metabolic profile of CML mice during disease progression, plasma samples were collected at three time points (days 0, 7, and 21) after drug withdrawal and subjected to untargeted metabolomics analysis (Fig. 1A). The results showed that lipid metabolites accounted for the largest proportion of identified metabolites, followed by amino acid metabolites and carbohydrate metabolites (Fig. 1B and Supplementary Table 1) [26]. Most of the metabolites were involved in cell membrane transport and signal transduction. Principal component analysis (PCA) showed a clear separation between the samples from days 0, 7, and 21 (Fig. 1C). The overall metabolic profile showed distinct reprogramming characteristics over time, as shown by metabolic heatmap analysis (Fig. 1D). Kyoto Encyclopedia of Genes and Genomes (KEGG) enrichment analysis of differential metabolites between day 0 and day 7 indicated that changes in the sphingolipid metabolism pathway were most significant at day 7 post-*BCR::ABL1* induction (day 7 vs day 0), followed by changes in the thiamine metabolism pathway and the tricarboxylic acid (TCA) cycle pathway. The TCA cycle pathway emerged as the most significant at day 21 post-*BCR::ABL1* induction, consistent with the Warburg effect observed in tumor cells (Fig. 1E) [27]. Further analysis revealed that changes in sphingolipid metabolism were primarily due to sphingosine (Sph) and sphingosine 1-phosphate (S1P), whereas changes in thiamine metabolism were largely attributed to thiamine pyrophosphate (TPP) (Fig. 1F). We then validated the levels of these three different metabolites in mouse plasma by quantitative mass spectrometry, and the results were consistent with the untargeted metabolomic analysis data (Fig. 1G).

In conclusion, our study describes the metabolic changes induced by BCR::ABL1 expression in a mouse model of CML. Given that alterations in sphingolipid and thiamine metabolism were most prominent at the early stage of *BCR::ABL1* expression, we hypothesize that dysregulation of these two metabolic pathways may be associated with the initiation of CML [28].

### Sphingolipid metabolism is more critical than thiamine metabolism for cell growth and death in CML

**Fig. 2:**
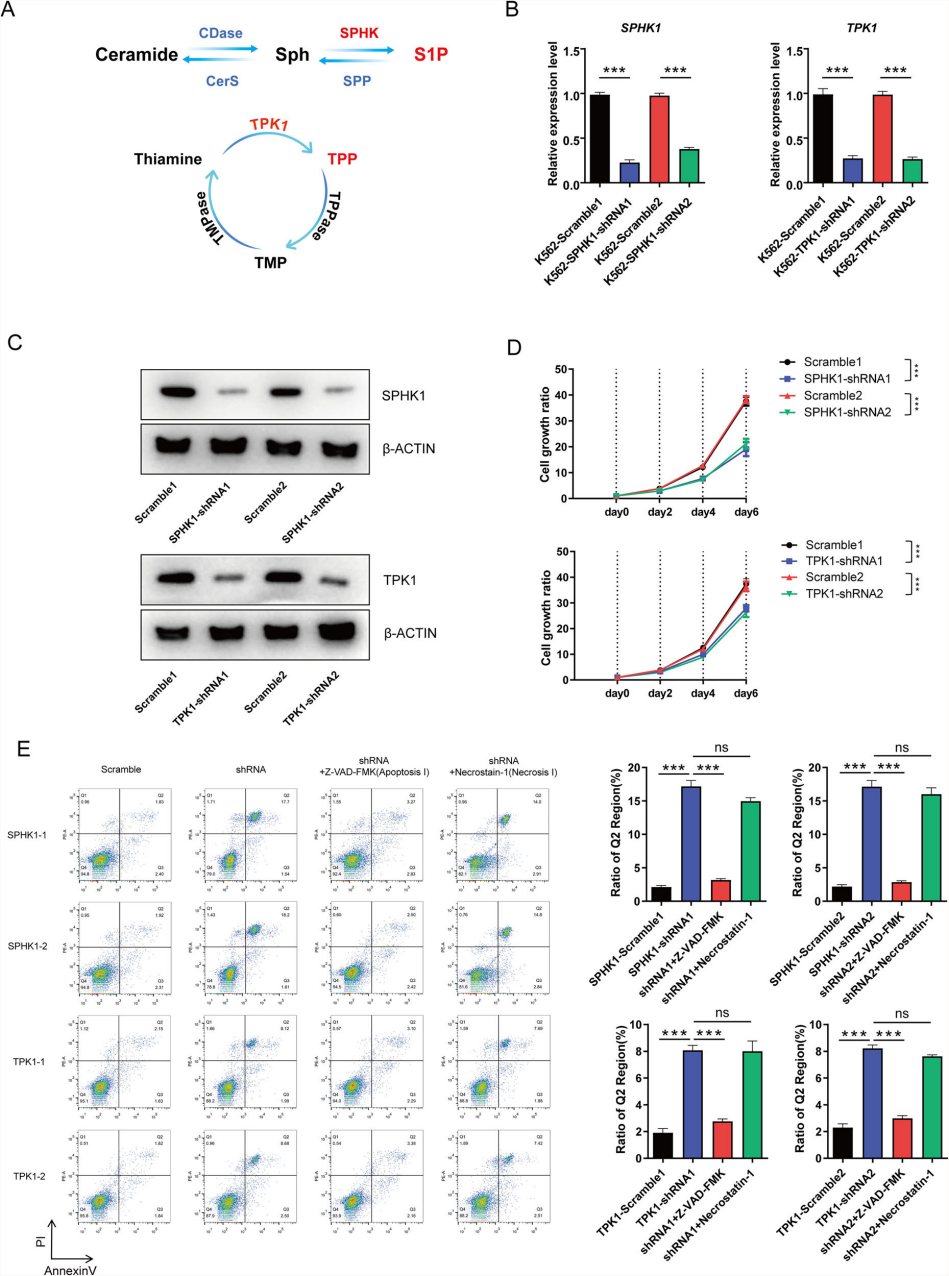
*SPHK1* knockdown has a more pronounced effect than *TPKI* knockdown on the growth and survival fate of K562 cells. **A** Diagram showing the sphingosine and thiamine metabolism pathways. **B** Validation of *SPHK1* and *TPK1* shRNA-mediated knockdown efficiencies in K562 cells using qRT-PCR (*n* = 3). **C** Validation of SPHK1 and TPK1 shRNA-mediated knockdown efficiencies in K562 cells using Western blot. **D** Detection of cell growth ability after *SPHK1* or *TPK1* knockdown (*n* = 3). **E** Flow cytometric analysis of cellular apoptosis after *SPHK1* or *TPK1* knockdown (*n* = 3). The bar graph data in Fig. 2 are presented as mean ± S.E.M.

It is known that the conversion of Sph to S1P is mainly regulated by the enzyme SPHK1, while the production of TPP is mainly facilitated by thiamine pyrophosphate kinase 1 (TPK1) (Fig. 2A) [29, 30]. To determine whether there is a mutual interaction between these two metabolic pathways in CML, we generated CML cell lines with shRNA-mediated *SPHK1* and *TPK1* knockdown, respectively. To ensure the reliability of the knockdown, we designed two separate shRNA sequences for each gene paired with the corresponding scramble sequence as a control, both of which demonstrated efficient knockdown, as confirmed by qPCR and Western blot analysis (Fig. 2B, C). We then assessed the effects of these two metabolic pathways on CML cell growth and death. The results showed that knockdown of both SPHK1 and TPK1 induced a decrease in cell growth and an increase in cell death, with the knockdown of SPHK1 being more pronounced (Fig. 2D, E) [31, 32], whereas no significant changes in the cell cycle were observed (Fig. S1). To further clarify the type of cell death induced by TPK1 and SPHK1 knockdown, K562 cells were treated after knockdown with an apoptosis inhibitor (Z-VAD-FMK) and a necrosis inhibitor (necrostatin-1) respectively. Our results showed that the apoptosis inhibitor restored the Q2 population, which increased most after knockdown, to near normal level, whereas the necrosis inhibitor did not show a significant rescue effect (Fig. 2E). Based on these findings, we suggest that the inhibition of cell growth induced by TPK1 and SPHK1 knockdown is mainly mediated by apoptosis, with SPHK1 knockdown having a more pronounced effect. Taken together, although both are early events in the metabolic reprogramming induced by *BCR::ABL1*, sphingolipid metabolism is more critical than thiamine metabolism for CML cell growth and death. Therefore, we decided to focus on the effects of sphingolipid metabolism alterations on the pathogenesis of CML.

We next asked whether the elevated S1P observed in CML was associated with other alterations in the sphingolipid pathways. To address this, we examined the mRNA and protein levels as well as the phosphorylation level of SPHK2 after *BCR::ABL1* overexpression and inhibition in 32D and K562 cells and no significant changes were observed, suggesting that SPHK2 is not a key regulator of SIP elevation (Fig. S2). We then examined the expression levels of S1P-cleaving enzyme (SGPL1) and phosphatases 1 and 2 (SGPP1 and SGPP2) to exclude the effect of reduced S1P catabolic pathway on S1P levels. The results showed that overexpression of BCR::ABL1 did not down-regulate the catabolic pathway of SIP in 32D cells, but instead significantly up-regulated the expression levels of SGPL1, SGPP1 and SGPP2. Consistent with this, K562 cells treated with imatinib showed significant downregulation of SGPL1, SGPP1 and SGPP2 (Fig. S2). These data suggest that the increase in S1P is mainly due to the increased SPHK1 kinase activity induced by BCR::ABL1 expression, and the upregulation of SGPL1, SGPP1 and SGPP2 upon BCR::ABL1 expression may be a feedback to the increase in SIP, acting as a brake.

### BCR::ABL1 upregulates S1P levels in multiple hematopoietic lineages in mice by increasing Sphk1 kinase activity

**Fig. 3:**
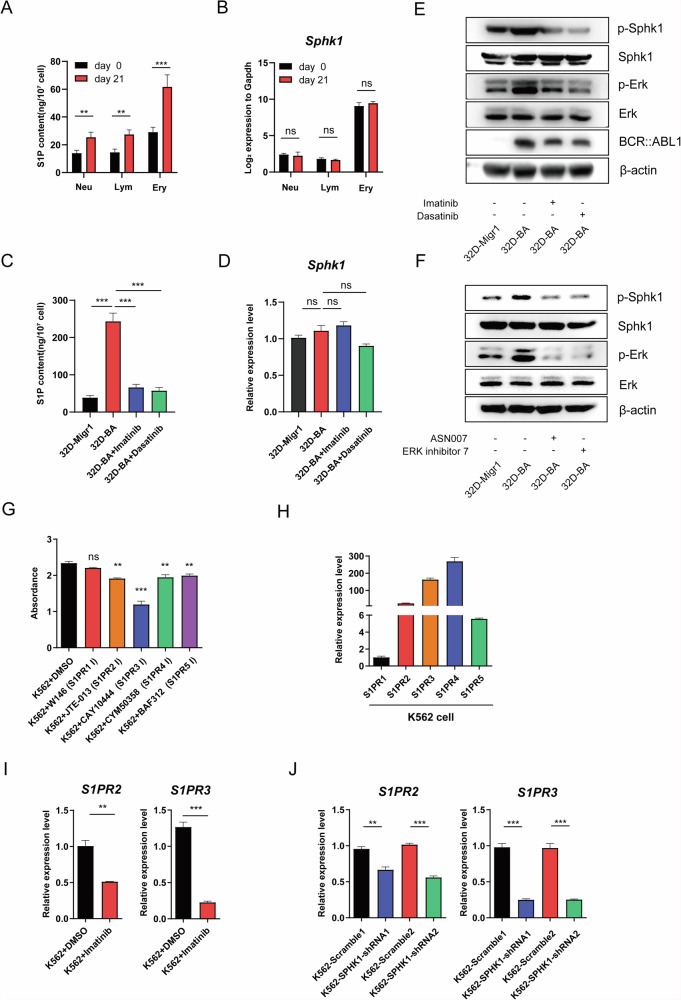
BCR::ABL1 Increased S1P levels in multiple hematopoietic lineages by regulating Sphk1 kinase activity. **A** Quantitative detection of S1P by mass spectrometry in different hematopoietic lineages in CML mice (*n* = 5). **B** qRT-PCR analysis of *Sphk1* mRNA levels in different hematopoietic lineages in CML mice (*n* = 3). **C** Quantitative detection of S1P by mass spectrometry in 32D-Migr1 and 32D-BA cells with and without TKI treatment (*n* = 5). **D** qRT-PCR analysis of *Sphk1* mRNA levels in 32D-Migr1 and 32D-BA cells with and without TKI treatment (*n* = 3). **E** Western blotting for the measurement of BCR::ABL1, Sphk1, p-Sphk1, Erk, and p-Erk levels in 32D-Migr1 and 32D-BA cells with or without TKI treatment. **F** Western blotting to measure Sphk1, p-Sphk1, Erk, and p-Erk levels in 32D-Migr1 and 32D-BA cells with and without ERK inhibitor treatment. **G** Cell viability of K562 cells detected by CCK-8 assay after treatment with S1PRs inhibitors (*n* = 3). **H** qRT-PCR analysis of *S1PR* expression levels in K562 cells (*n* = 3). **I** qRT-PCR analysis of *S1PR2* and *S1PR3* mRNA levels in K562 cells with and without TKI treatment (*n* = 3). **J** qRT-PCR analysis of *S1PR2* and *S1PR3* mRNA levels in K562 cells with and without *SPHK1* knockdown (*n* = 3). The bar graph data in Fig. 3 are presented as mean ± S.E.M.

SPHK1 is specifically expressed in the erythroid lineage and SPHK1 overexpression has been identified as an oncogenic event in the progression of erythroleukemia [33]. Given that the K562 cell line was derived from a CML patient who developed acute erythroleukemia [34], we hypothesized that the BCR::ABL1-SPHK1-S1P regulatory axis might be erythroid-specific. To address this issue, bone marrow (BM) samples were collected from Scl/tTA-*BCR::ABL1* transgenic mice on days 0 and 21 after BCR::ABL1 induction. Granulocytes, lymphocytes, and erythroid cells were isolated by flow sorting, and SIP levels were determined by quantitative mass spectrometry. Surprisingly, a significant increase in SIP levels was observed in all three cell types at day 21 compared to day 0, but erythroid cells still contribute the most to the increase with the highest basal S1P level in these cell types (Neu-day 0 (13.936, ±1.932 (SD), ±0.864 (SEM), *n* = 5); Lym-day 0 (14.588, ±2.273 (SD), ±1.017 (SEM), *n* = 5); Ery-day 0 (29.150, ±3.317 (SD), ±1.483 (SEM), *n* = 5); Neu-day 21 (25.436, ±3.643 (SD), ±1.629 (SEM), *n* = 5); Lym-day 21 (27.464, ±3.164 (SD), ±1.415 (SEM), *n* = 5); Ery-day 21 (61.638, ±8.053 (SD), ±3.601 (SEM), *n* = 5) (Fig. 3A). Previous studies have suggested that BCR::ABL1 can upregulate Sphk1 expression [20]. However, *Sphk1* transcript levels remained unchanged after *BCR::ABL1* induction in these cell types (Fig. 3B), indicating that the elevated SIP was not due to transcriptional changes in *Sphk1*. To verify this, *BCR::ABL1* was introduced into 32D cells [35]. As expected, *BCR::ABL1* expression significantly increased S1P levels, which could be reversed by various tyrosine kinase inhibitors (TKIs) (Fig. 3C). Consistent with the findings in CML mice, Sphk1 expression levels in 32D-BA cells remained unchanged and unresponsive to TKI treatment (Fig. 3D, E). However, BCR::ABL1 upregulated the phosphorylation levels of both Sphk1 and Erk, which were decreased by TKI treatment (Fig. 3E). Furthermore, treatment with Erk activity inhibitors did not alter Sphk1 protein levels but significantly reduced its phosphorylation (Fig. 3F). These results demonstrate that BCR::ABL1 exerts a pervasive influence on sphingolipid metabolism in multiple hematopoietic lineages in mice. The increase in SIP levels was attributed to increased kinase activity rather than changes in Sphk1 expression. In addition, BCR::ABL1 may enhance Sphk1 kinase activity by regulating the phosphorylation level of Erk.

To further elucidate the downstream pathway mediating the influence of SIP on CML progression, we examined the effects of specific inhibitors of five S1P receptors (S1PR1-5) on CML cell growth and found that the S1PR3 inhibition group showed the most significant decrease in cell growth. We also analyzed the effect of BCR-ABL expression and SPHK1 knockdown on S1PR1-5 expression levels and found that the most pronounced changes in receptor expression also occurred in S1PR3, suggesting that elevated S1P may trigger S1PR signaling, particularly SIPR3 signaling (Fig. 3G–J, Fig. S3).

### Sphingolipid metabolism plays a pivotal role in metabolic remodeling in CML

**Fig. 4:**
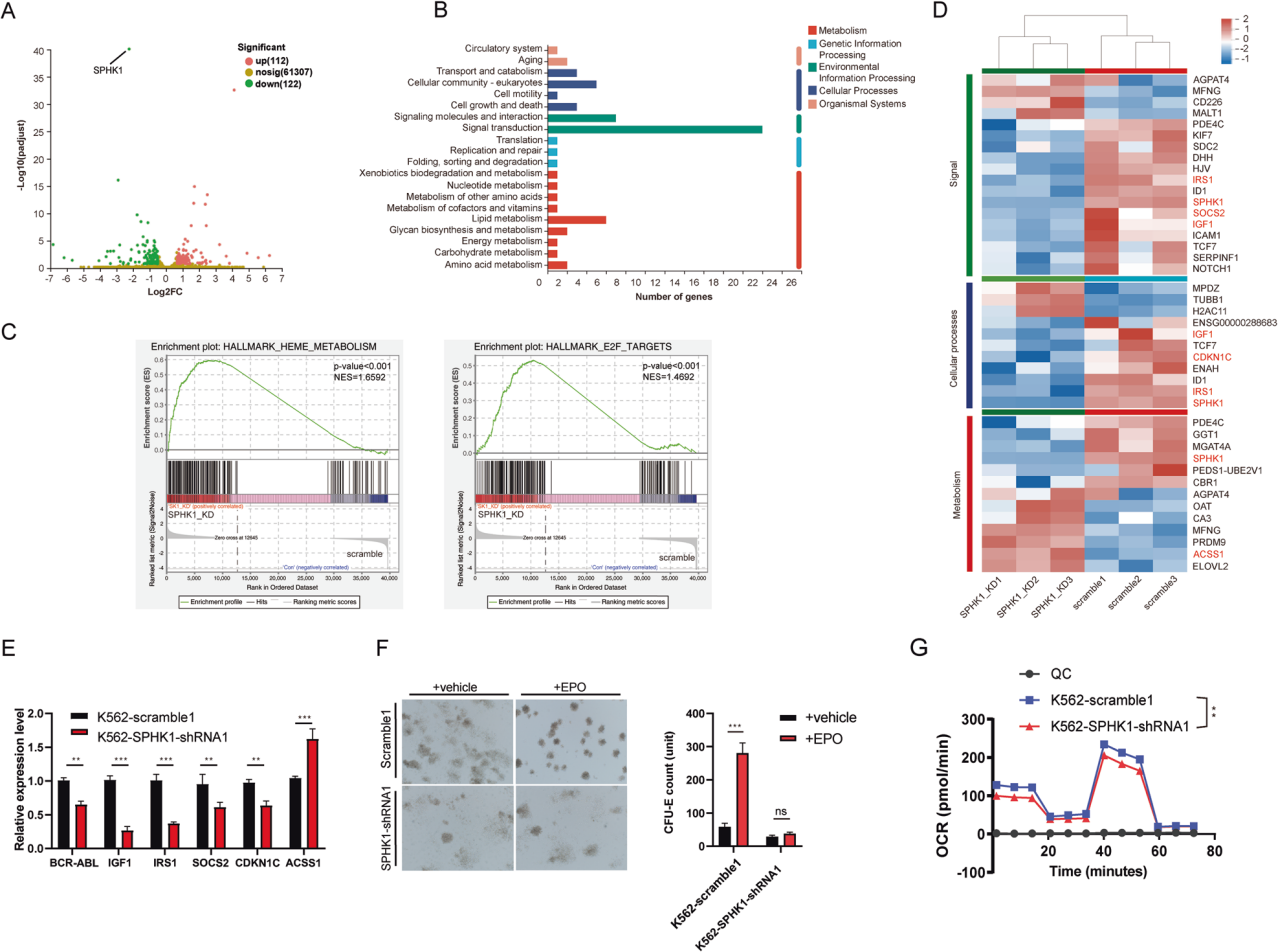
*SPHK1* knockdown alters metabolic pathways in K562 cells. **A** Volcano plot of differentially expressed genes (DEGs) (log2FC ≥ 1 or log2FC ≤ -1 and *P*-value ≤ 0.05). **B** KEGG enrichment analysis of DEGs. **C** GSEA analysis showing the top discrepant hallmark gene sets. **D** Heatmap analysis of genes enriched in signal transduction, cellular processes and metabolic pathways. **E** qRT-PCR validation of DEGs (*n* = 3). **F** Erythroid colony forming unit assay (*n* - 3). **G** Mitochondrial stress test by Seahorse assay (*n* = 6). The bar graph data in Fig. 4 are presented as mean ± S.E.M.

S1P is involved in numerous physiological processes, including signal transduction, regulation of vascular function, modulation of the immune response, tissue repair and regeneration, lipid metabolism, and other biological functions [16, 17]. To gain deeper insight into the impact of sphingolipid metabolism on CML cells, we performed RNA-seq analysis of K562 cells with shRNA-mediated knockdown of *SPHK1*. The volcano plot of differentially expressed genes (DEGs) showed 112 up-regulated and 122 down-regulated genes, with *SPHK1* showing the most statistically significant difference among all DEGs (Fig. 4A). These genes were particularly enriched in pathways related to genetic and environmental information processing, cellular processes, and metabolism, with lipid metabolism being the most significantly enriched process among the metabolic pathways (Fig. 4B) [36]. Gene set enrichment analysis (GSEA) further revealed significant negative enrichment in gene sets associated with heme metabolism and E2F targets following *SPHK1* knockdown (Fig. 4C) [37]. Further heatmap analysis was performed with DEGs associated with signal transduction, cellular processes and metabolic pathways. Notable genes such as *IGF1*, *IRS1*, and *SPHK1* were found in multiple classifications simultaneously. Among these DEGS, *SOCS2*, *ACSS1*, and *CDKN1C* are associated with cellular processes and heme metabolism (Fig. 4D) [38–40]. RT-qPCR validation confirmed differential expression consistent with the transcriptomic data. Of particular note, a significant reduction in *BCR::ABL1* transcript expression was observed upon *SPHK1* knockdown (Fig. 4E), suggesting a feedback mechanism between BCR::ABL1 and SPHK1.

In view of the GSEA indicating a dysregulation of heme metabolism following *SPHK1* knockdown, an erythrocyte colony formation assay was performed. Indeed, our results showed that the ability of K562 cells to differentiate into erythrocytes was reduced (Fig. 4F) [41]. In addition, KEGG analysis revealed that some of the DEGs were enriched in the energy metabolism pathway (Fig. 4B). To confirm this result, we evaluated the effect of *SPHK1* knockdown on mitochondrial respiration in K562 cells using the Seahorse assay. A significant reduction in both the basal oxygen consumption rate (OCR) and the maximal OCR following FCCP stimulation was observed in the *SPHK1* knockdown group, indicating an impaired mitochondrial respiratory capacity (Fig. 4G). These findings highlight the pivotal role of sphingolipid metabolism in BCR::ABL1-induced metabolic reprogramming.

### Pharmacological targeting of sphingolipid metabolism attenuates CML development

**Fig. 5:**
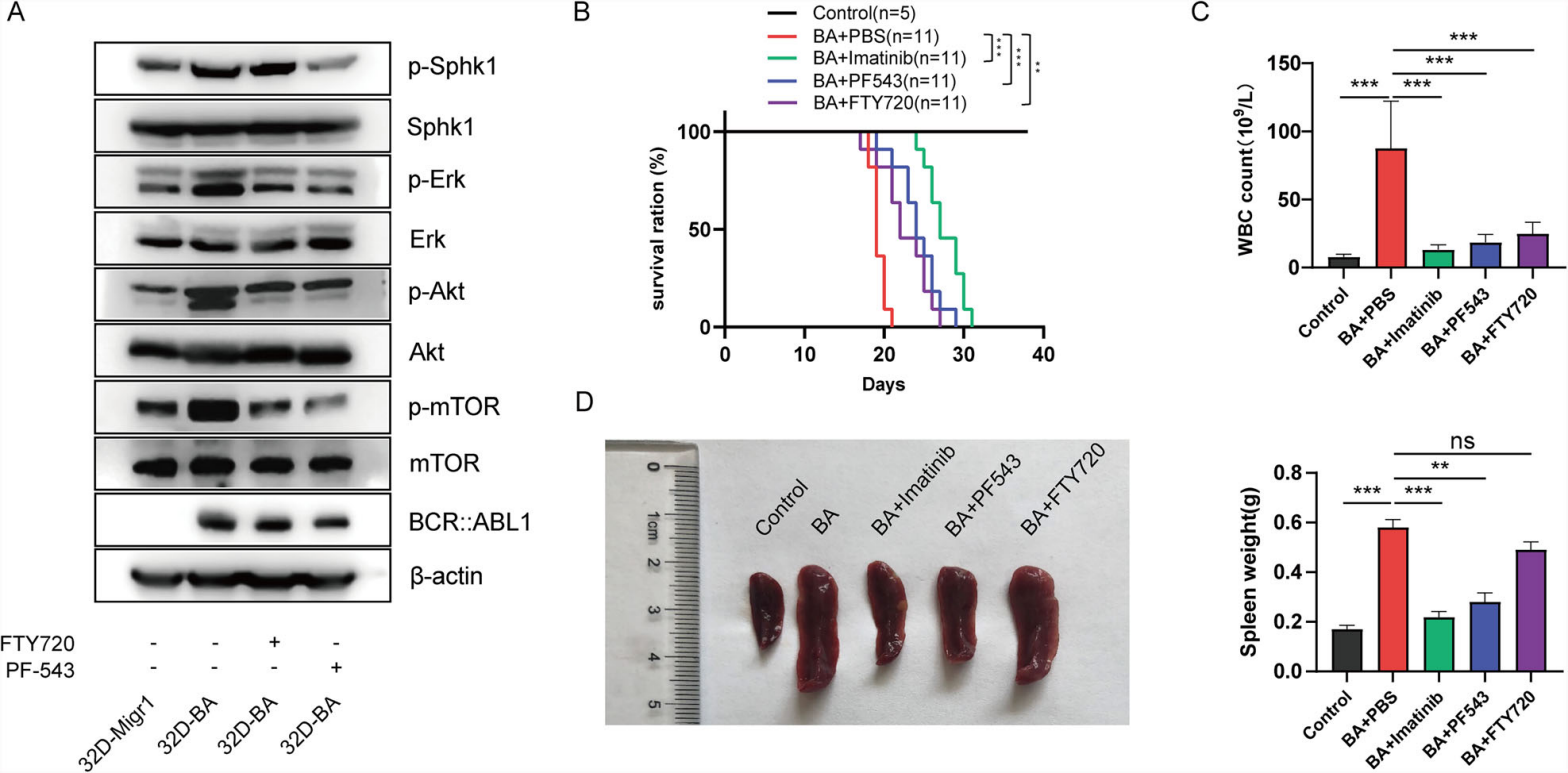
Inhibition of sphingolipid metabolism attenuates CML development. **A** Western blotting measuring protein and phosphorylation levels of BCR::ABL1, Sphk1, Erk, mTOR and Akt in 32D-Migr1 and 32D-BA cells treated with SPHK1 kinase and SIP receptor inhibitor. **B** Survival curve analysis of CML-BMT mice treated with SPHK1 kinase and SIP receptor inhibitor, imatinib was used as a positive control (*n* = 12). **C** Statistical analysis of peripheral white blood cells in CML-BMT mice after one week of drug treatment. **D** Analysis of spleen changes in CML-BMT mice after treatment. The bar graph data in Fig. 5 are presented as mean ± S.E.M.

A previous in vitro study has demonstrated the anti-leukemic activity of the SPHK1 inhibitor [42], but its effect in vivo remains unclear. Therefore, we selected the SPHK1 kinase inhibitor PF-543 and the S1P receptor inhibitor FTY720 for in vivo treatment of CML mice [43]. First, we evaluated their inhibitory effects on the SPHK1-S1P pathway in 32D-BA cells. As expected, PF-543 significantly reduced the phosphorylation level of SPHK1, whereas the effect of FTY720 was less pronounced. Both inhibitors significantly reduced the phosphorylation levels of ERK, mTOR, and Akt, indicating effective inhibition on the activation of the SPHK1-S1P pathway (Fig. 5A).

The in vivo effects of these inhibitors were then evaluated in a BCR::ABL1 retrovirus-induced CML mouse model, using imatinib as a positive control. Compared to the PBS-treated group, mice treated with the SPHK1 kinase inhibitor PF-543 and the S1P receptor inhibitor FTY720 exhibited significantly prolonged survival (median survival: PF543 (24.27 days) >FTY720 (22.64 days) >PBS (19.27 days)) (Fig. 5B), reduced peripheral blood leukocyte counts (Fig. 5C), and decreased spleen size (Fig. 5D). These findings suggest that pharmacological targeting of sphingolipid metabolism may attenuate the development of CML [44].

## Discussion

Metabolic reprogramming is recognized as a hallmark of cancer, with increasing research interest in this area. Metabolic changes provide cancer cells with advantages in energy use, promoting proliferation, metastasis, and drug resistance. The identification of key enzymes in interconnected metabolic pathways holds great promise for tumor treatment [45, 46].

To date, studies of metabolic alterations and regulatory mechanisms in CML have largely focused on in vitro models, with limited understanding of metabolic reprogramming during CML initiation and progression in vivo. In this study, using Scl/tTA-*BCR::ABL1* mice as a model, we analyzed changes in the metabolic profile at different stages following *BCR::ABL1* induction and found a substantial increase in sphingosine metabolism during CML initiation. Knockdown of *SPHK1* not only inhibits cell growth and induces cell death but also disrupts key metabolic processes, such as lipid, heme, and energy metabolism. Importantly, pharmacological inhibition of the sphingosine signaling pathway effectively attenuated CML progression. These findings highlight the importance of sphingosine metabolism in BCR::ABL1-induced metabolic reprogramming and its role in CML pathogenesis.

It is known that the production of S1P can result from various alterations in the sphingolipid metabolism. Is the increase in S1P observed in CML associated with alterations other than SPHK1? Here, we excluded the effect of SPHK2 activity on SIP levels by examining the expression and phosphorylation levels of SPHK2 after *BCR::ABL1* overexpression and inhibition. Notably, with respect to the S1P catabolic pathway, we observed that BCR::ABL1 overexpression significantly up-regulated the expression levels of SGPL1, SGPP1 and SGPP2 in 32D cells. Consistent with this, a significant downregulation of SGPL1, SGPP1 and SGPP2 expression levels was also observed in K562 cells treated with imatinib (Fig. S2). These results suggest that the increase in S1P is mainly due to the increase in SPHK1 kinase activity induced by BCR::ABL1 expression. The up-regulation of SGPL1, SGPP1 and SGPP2 upon BCR::ABL1 expression may be a feedback of increased SIP.

In this study, the increase in S1P in BM subsets after BCR::ABL1 induction is significant but moderate. Similar to our results, Tang et al. reported that serum S1P levels in lung cancer patients were about 2-fold higher than those in the normal population, as well as about 2-fold higher during radiotherapy than during non-radiotherapy [47], suggesting that modest changes in S1P are sufficient to induce pathological effects in vivo. We speculate that dramatic increases in S1P in vivo may be lethal to an organism, and that the catabolism of S1P in vivo may be better regulated than in the cell lines, thus maintaining S1P levels at moderate levels. Indeed, as mentioned above, the key enzymes in the S1P catabolic pathway were upregulated following BCR::ABL1 expression, which may act as a brake (Fig. S2).

Previous studies have shown that BCR::ABL1 activates *SPHK1* transcription through downstream signaling molecules, and SPHK1-S1P-S1PR2 in turn enhances BCR::ABL1 stability by regulating protein phosphatase 2A [22, 25]. Here we have shown that BCR::ABL1 enhances SPHK1 kinase activity by regulating the phosphorylation level of ERK, thereby aberrantly activating sphingolipid metabolism and establishing a positive feedback loop with *BCR::ABL1* transcription. Given that dysregulation of sphingolipid metabolism is also present in other hematopoietic malignancies and solid tumors, this novel feedback mechanism may also be applicable to the interaction between SPHK1 and other tumor drivers. In addition, we evaluated the expression of S1P receptors (S1PR1-5) and the consequences of S1P receptor inhibition on K562 cell growth to further elucidate the downstream pathway mediating the influence of SIP on CML progression. The results showed that S1PR3-specific inhibitor exerted the strongest inhibitory effect on K562 cell growth, in line with this, BCR::ABL1 expression and SPHK1 knockdown also resulted in the most pronounced changes in receptor expression, suggesting that elevated S1P may trigger S1PR signaling, particularly SIPR3 signaling (Fig. 3G–J, Fig. S3).

In addition to sphingolipid metabolism, we observed that thiamine metabolism was also significantly increased at the early stage of CML onset. However, compared to SPHK1 knockdown in vitro, TPK1 knockdown has a lesser effect on CML cell growth and death. The potential role of thiamine metabolism in CML pathogenesis in vivo could not be assessed due to the lack of TPK1 inhibitors. The thiamine metabolic pathway, which is crucial for energy metabolism, involves a number of processes, including thiamine uptake, transport, and phosphorylation [48]. TPP, the active product catalyzed by TPK1, serves as a coenzyme for metabolic enzymes like pyruvate dehydrogenase and α-ketoglutarate dehydrogenase, which are critical for the regulation of the TCA cycle, cell proliferation, survival, and chemotherapy resistance in tumors [49]. Notably, we observed a significant upregulation of TCA metabolism during CML development, which may be associated with excessive TPP elevation. Although both sphingolipid and thiamine metabolism are aberrantly activated at the onset of CML, disruption of one pathway does not affect the other, suggesting that these two pathways play independent roles in CML pathogenesis.

In conclusion, this study provides insights into the in vivo metabolic profile of CML and highlights the dominant role of sphingolipid metabolism in BCR::ABL1-induced metabolic reprogramming.

## Supplementary information

Supplementary Table and Figure legends

Supplementary table 1

Supplementary Figure 1

Supplementary Figure 2

Supplementary Figure 3

Western Blot pictures

## Data Availability

All sequencing data and metabolomics data included in this study are available in the National Omics Data Encyclopedia (NODE) (accession number: OEP00005840). All data generated or analyzed in this study are included in this published article.
