# Supplementary material for: Dysregulation of sphingolipid metabolism contributes to the pathogenesis of chronic myeloid leukemia
Source: Cell Death Dis. 2025 Apr 13;16(1):282. doi: 10.1038/s41419-025-07594-0 (PMC11993578; doi:10.1038/s41419-025-07594-0)
Supplement: Supplementary file 1 — Supplementary Table and Figure legends [file 41419_2025_7594_MOESM1_ESM.doc]

SUPPLEMENTARY TABLE AND FIGURE LEGENDS

Supplementary Table. 1

Complete list of differentially expressed metabolites under BCR::ABL induction

Supplementary Fig. 1 Knockdown of SPHK1 and TPK1 does not significantly affect the cell cycle in K562 cells.

1. Flow cytometry analysis of cell cycle after SPHK1 or TPK1 knockdown (n=3).

The bar graph data in supplementary Fig. 1 are presented as mean±S.E.M.

Supplementary Fig. 2 BCR::ABL1 Influences the expression of S1P-Related Enzymes

1. qRT-PCR analysis of *Sphk2*, *Sgpl1, Sgpp1* and *Sgpp2* mRNA levels in 32D-Migr1 and 32D-BA cells (n=3).
2. Western blotting for measurement of Sphk2, Sgpl1, Sgpp1 and Sgpp2 protein levels in 32D-Migr1 and 32D-BA cells (n=3).
3. qRT-PCR analysis of *SPHK2*, *SGPL1, SGPP1* and *SGPP2* mRNA levels in K562 cells with or without TKI treatment (n=3).
4. Western blotting for measurement of SPHK2, SGPL1, SGPP1 and SGPP2 protein levels in K562 cells with or without TKI treatment (n=3)

The bar graph data in supplementary Fig. 2 are presented as mean±S.E.M.

Supplementary Fig. 3 S1PR1/S1PR4/S1PR5 showed no significant changes in K562 cells

(A) qRT-PCR analysis of *S1PR1, S1PR4* and *S1PR5* mRNA levels in K562 cells with or without TKI treatment (n=3)

(B) qRT-PCR analysis of *S1PR1, S1PR4* and *S1PR5* mRNA levels in K562 cells with or without *SPHK1* knockdown (n=3)

The bar graph data in supplementary Fig. 3 are presented as mean±S.E.M.
