# Supplementary figures and images for: Dysregulation of sphingolipid metabolism contributes to the pathogenesis of chronic myeloid leukemia
Source: Cell Death Dis. 2025 Apr 13;16(1):282. doi: 10.1038/s41419-025-07594-0 (PMC11993578; doi:10.1038/s41419-025-07594-0)

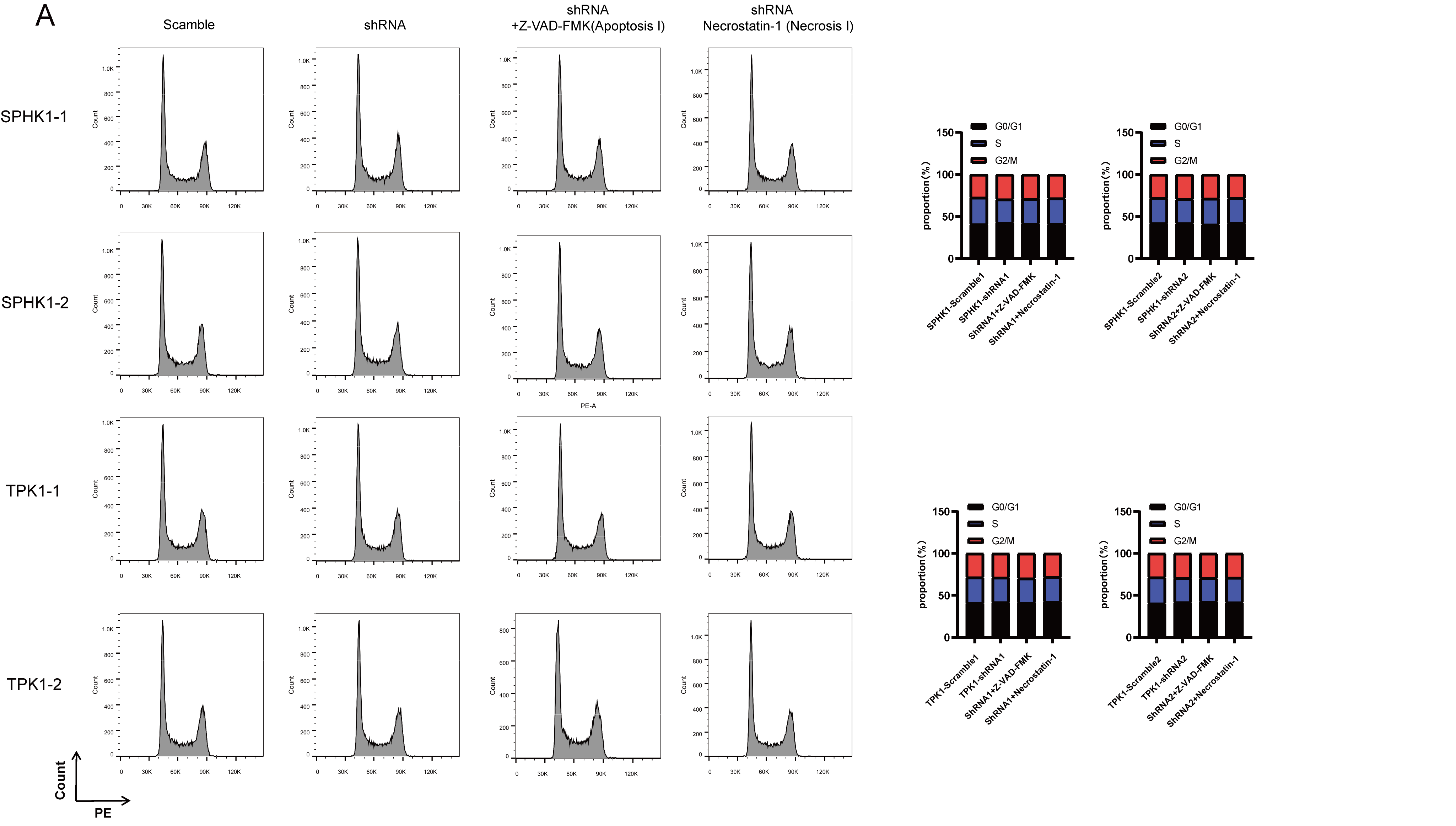

Supplement: Supplementary file 3 — Supplementary Figure 1 [file 41419_2025_7594_MOESM3_ESM.tif]

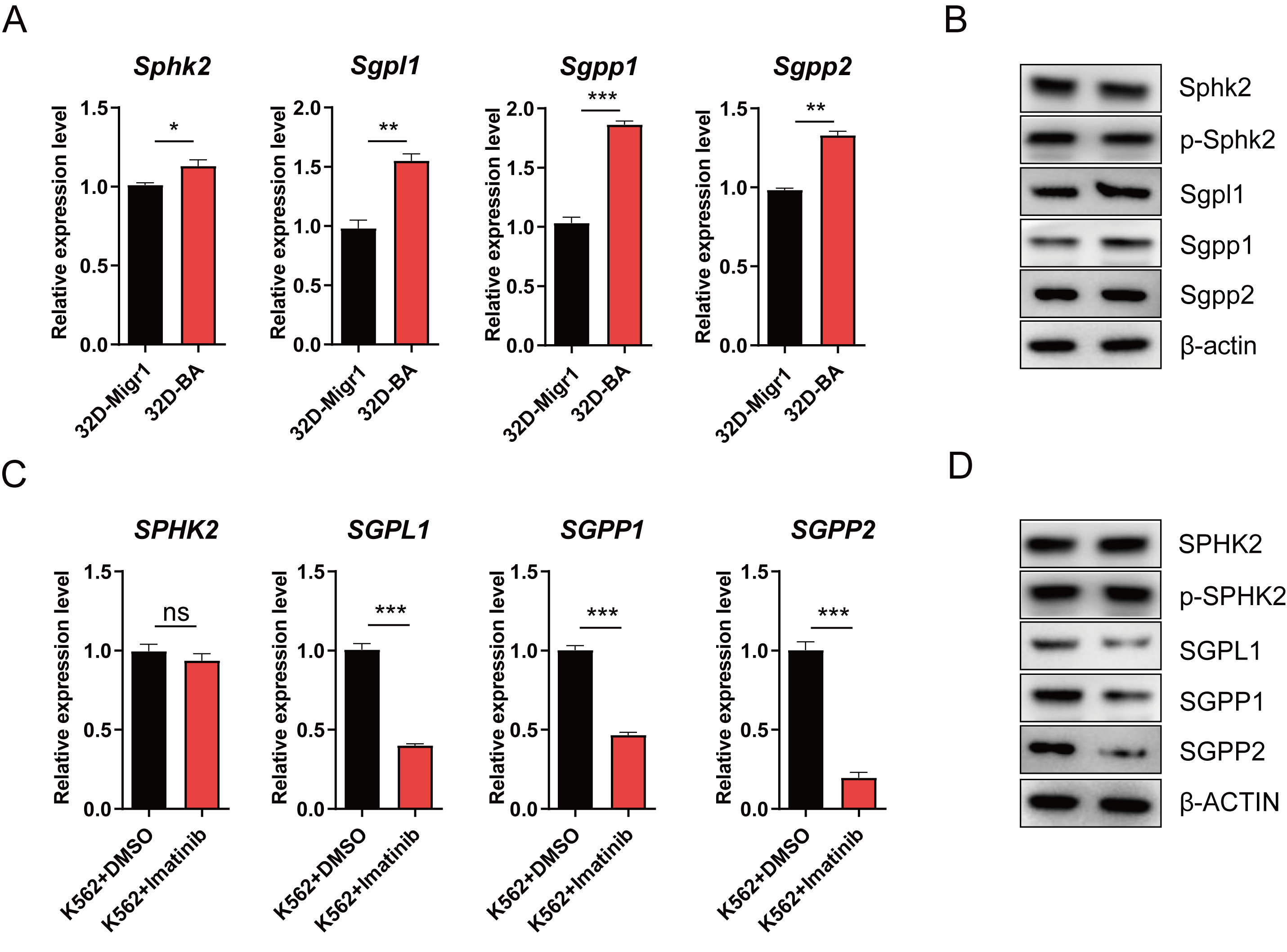

Supplement: Supplementary file 4 — Supplementary Figure 2 [file 41419_2025_7594_MOESM4_ESM.tif]

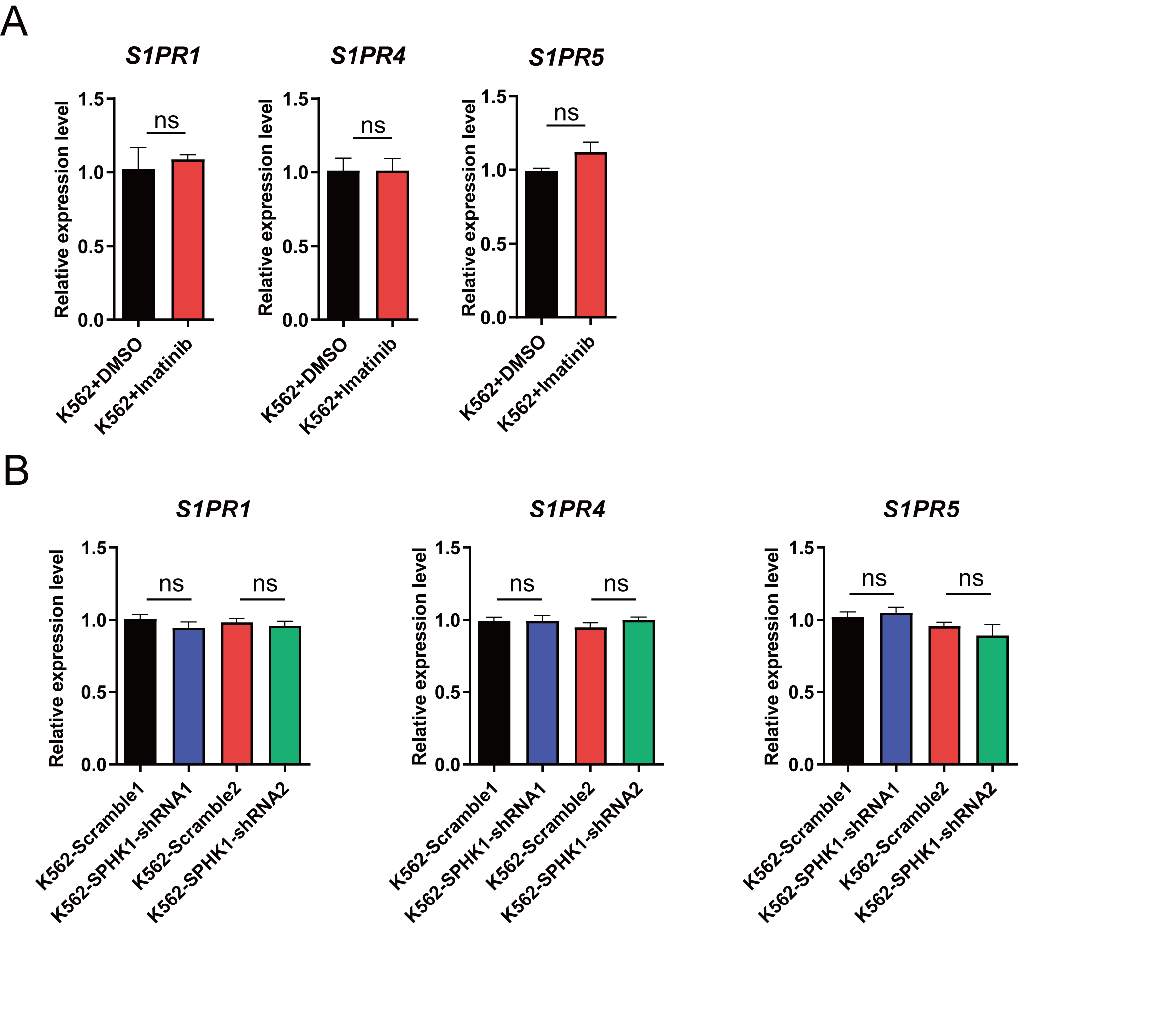

Supplement: Supplementary file 5 — Supplementary Figure 3 [file 41419_2025_7594_MOESM5_ESM.tif]
